# Cultural and age differences in beliefs about depression: British Bangladeshis vs. British Whites

**DOI:** 10.1080/13674676.2013.785710

**Published:** 2013-04-16

**Authors:** Alastair McClelland, Shopnara Khanam, Adrian Furnham

**Affiliations:** Division of Psychology and Language Sciences, University College London, UK

**Keywords:** age, culture, depression, lay theories, British, Bangladeshis

## Abstract

This study examines beliefs about depression as a function of ethnic background (British Bangladeshis vs. British Whites) and age. A total of 364 participants completed a 65-item questionnaire, containing general questions regarding depression and anti-depressive behaviour; the causes of depression, and treatments for depression. The hypotheses were broadly supported; there were significant interactions between ethnicity and age, which generally revealed an increasingly negative attitude towards depression with increasing age amongst British Bangladeshis. Older British Bangladeshis believed depression was an illness that brought a sense of shame and loss of dignity to the individual and his or her family, and they also favoured a lay referral system for sufferers. They also had more superstitious beliefs about depression than both younger British Bangladeshis and British Whites. A pattern of increasing negativity with increasing age was not evident amongst the British Whites, but older individuals in both groups tended to believe that depression was not helped by psychological intervention. The attitudes towards depression in the young was similar (and generally positive) in both ethnic groups. These findings highlight the necessity to provide more culturally sensitive and accessible services for migrant communities – particularly amongst older individuals.

## Introduction

This study concerns cultural differences in beliefs about depression which is a topic that has been extensively explored (e.g., Lynch, Kendrick, Moore, Johnson, & Smith, 2006; Waite & Killian, 2008). It also focuses on age differences in attitudes to treatment (Gonzalez, Alegria, Prihoda, Copeland, & Zeber, 2011). Kirmayer (2001) has argued that the individual's explanatory models, coping styles, help-seeking behaviour and social response to disability are all influenced by his or her culture. There is substantial research indicating a difference in the access to, utilisation of, and treatments prescribed by mental health services between British Whites, and Black and Asian ethnic minorities (Lloyd & Moodley, 1992).

The Southeast Asian community views depression as “self-indulgent” and there is little tolerance for cognitions of the self (Furnham & Malik, 1994). Karasz (2005) comparing illness presentation between South Asians and White Americans, found that the South Asians interpreted the symptoms of depression in situational terms – as an emotional reaction as opposed to a pathogenic state – and were unable to label the illness. Aspect of the concept of depression may be unique to Western cultures (Tsai & Chentsova-Dutton, 2002). However, other cultures do appear to differ in such expectations. Whether depression is experienced or expressed in psychological, emotional or physical terms is likely to be a reflection of the cultural background of the individual (Desjarlais, Eisenberg, Good, & Kleinman, 1995). Members of ethnic minorities, particularly Asians, usually only choose to utilise mental health care services when they think that their altered state of functioning is related to their physical well-being (Bhui & Bhugra, 2002).

Cochrane's (1979) community survey found that there was an under-representation of British Asians in psychiatric statistics, particularly for affective disorders such as depression. Asians are known to frequently present in primary care, but not with mental illness (Gilliam, Jarman, White, & Law, 1989). Furthermore, primary care research in the UK reveals that although South Asians are found to make *more* frequent visits to their general practitioners than the British Whites, they are *less* likely to have their psychological difficulties (and particularly depression) identified (Bhugra & Mastrogianni, 2004).

Furnham and Malik (1994) provided several possible explanations for this under-representation. Firstly, it could imply that depression is a Western phenomenon and that Asians are psychologically healthier. Marsella and White (1982) found that symptoms commonly manifested by sufferers from the West are rare or non-existent in non-Western cultures. Secondly, the somatisation hypothesis may explain the under-representation of mental illness amongst British Asians. It has been suggested that Asians living in the UK are reluctant to disclose psychological problems to health professionals (Sheikh & Furnham, 2000). Thirdly, there is clinical evidence for Asians who become depressed to commonly present with somatic problems; usually complaints of widespread pain, headaches, and difficulty in breathing (Rack, 1982). In a review of the research literature, Kirmayer and Young (1998) concluded that somatisation may be ubiquitous – but is more commonly found in individuals from a non- Western cultural background. In a community survey in the UK, Nazroo (1997) found that there were lower rates of depression amongst Asian migrants, including British Bangladeshis, compared with their British White counterparts. Nevertheless, this may simply be due to a failure to recognise depression by both the health professional and the Asian sufferers themselves. Donovan (1986) suggested that Asian women are unable to recognise depression as an illness because they were unable to dissociate symptoms of depression from the feeling of loneliness that accompanied their migration to Britain; hence depression was perceived as part of an everyday life experience.

The beliefs of British Asian migrants in the UK are influenced by the values of both their native culture and the host culture (Helman, 1990); however there is evidence for traditional cultural beliefs to be more deeply embedded and structured than those that exist in the Western culture (Skeikh & Furnham, 2000). In Western countries the public attribute depression mainly to social-environmental factors, and biological causes are viewed as less important (Jorm et al., 1997). This suggests that those who attribute mental illness to factors that are outside the control of the individual, i.e., biological factors, will have a less negative reaction towards the mentally ill, whereas those who attribute the illness to the individual themselves are less likely to interact with the mentally ill. Dietrich et al. (2004) found a positive relationship between blaming the mentally ill individual and increasing the social distance from the mentally ill. However the study also found a desire to increase social distance with those who had biologically based causal beliefs regarding mental illness.

In non-Western cultures, there is a tendency to attribute a supernatural force, such as black magic and possession by evil spirits as the underlying cause for mental illness (Jorm, 2000). The use of religious or spiritual healers, who offer culture-specific approaches to mental illness and treatment, is an established form of treatment within the Asian communities. Asians living in the UK are known to use such traditional healers (Hussain & Cochrane, 2004). More specifically, there is a strong belief in the existence of Jinn (beings who cannot be seen but are able to cause harm to humans through possession) and the “evil eye” (the power of envy which can cause both mental and physical ill-health) in the Muslim community. In interviews conducted with 40 Bangladeshis living in East London, Dein, Alexander, and Napier (2008) investigated the role played by Jinn in explaining both psychological and physical illness. They found that mental illness was commonly attributed to Jinn possession, particularly amongst the older and less well-educated individuals, who would seek assistance from traditional healers. Khalifa, Hardie, Latif, Jamil, and Walker (2011) found that in a sample of Muslims living in Leicester, 80% believed in Jinn, 74% in the evil eye and 65% in black magic, with a majority believing that Jinn and the evil eye could cause mental illness. Khalifa et al. (2011) also reported that majority of the participants in their study believed that religious figures – rather than doctors – should be the treating authority for afflictions caused by Jinn, black magic and the evil eye (see also Khalifa & Hardie, 2008, for illustrative case studies) Furthermore, cognitive factors (particularly religion and prayer) have been reported as important in managing depression by Pakistani Muslim women (Cinnirella & Loewenthal, 1999).

A number of studies have demonstrated the impact cultural conceptions of mental illness have on help-seeking and the way in which the mentally ill are treated by health professionals and the public (Furnham & Chan, 2004). Asian cultures place more emphasis on the lay referral system, particularly turning to a family member for help (Furnham & Malik, 1994). This is consistent with Karasz's (2005) findings that South Asian women predominantly focused on the family – and particularly their husbands – for support. Angermeyer, Matschinger, and Riedel-Heller (1999) found that the public favoured lay support in the treatment of depression, whereas mental health professionals were considered more helpful in the treatment of schizophrenia. This may be due to people having differing perceptions of the aetiology of these mental illnesses, with depression being viewed as more socially constructed, whereas schizophrenia is viewed as having a biological cause, requiring medical assistance. The seeking of professional help is relatively rare in many non-Western societies, and among immigrant and minority groups in the West (Sussman, Robins, & Earls, 1987); moreover ethnic minority members are less likely to utilise voluntary specialty mental health services (Ying & Miller, 1992).

The Asian culture has a tendency to conceal mental illness from the wider community, as the reputation of the family is at stake (Furnham & Chan, 2004). Asian communities seem to attach greater stigma to mental illness than that of their British White counterparts (Hussain & Cochrane, 2004). Qureshi (1988) suggested that one reason for this is that in Asian cultures, the disclosure of mental illness in the family reduces the prospects of an arranged marriage. Thus the interest of the family supersedes individual interests. This may lead to a preference for private coping strategies.

The present study explores the attitudes of both the young and older generation of British Bangladeshis compared to British Whites, and will further develop our understanding of cultural differences. It is hypothesised that overall, the British Bangladeshis will have more conservative and negative attitudes towards depression than British Whites but this effect will be moderated by age; the younger British Bangladeshis will have similar beliefs and attitudes towards depression as British Whites. It was also predicted that Older British Bangladeshis will be more inclined to seek help from their family and to make use of “lay referral systems” than British Whites, and that British Bangladeshis will have more superstitious beliefs about depression than the British Whites.

## Method

### Sample

A total of 364 participants completed the questionnaire, of which 190 were British Bangladeshis living in East London, and 174 were British Whites. The majority of the younger participants were recruited from University of London and others through personal contact. The majority of the older participants were recruited from two local community centres (British Bangladeshis from the Bromley-by-Bow Community Centre (London borough of Tower Hamlets) and British Whites from the Froud Community Centre (London borough of Newham). The remainder of the participants were recruited via the local libraries in the London borough of Newham, and a small number through personal contact. Most of the questionnaires were completed in the presence of one of the researchers, but a number were distributed and then collected after completion. Participation in the study was entirely voluntary, and each participant gave verbal consent prior to completing the questionnaire. The study was granted ethical approval by the University College London Medical School.

### Measures

The questionnaire used in the study was constructed from questions used in three previous studies; (Furnham & Chan, 2004; Furnham & Kuyken, 1991; Rippere, 1977). In addition, nine new questions concerning superstitious beliefs and family values were constructed and included in the questionnaire. The questionnaire was piloted using 10 British Bangladeshi and six British White participants. The first section had 22 questions on people's beliefs about depression and anti-depressive behaviour. The second section consisted of 23 questions on causal explanations for depression. The third section consisted of 20 questions, and related to the treatment of depression. The last part of the questionnaire consisted of demographic details about the participants. There were also questions relating to the participant's medical history with respect to depression and other mental health problems, and questions tapping their experience of mental health problems suffered by acquaintances. Finally, there was a rating scale to assess how religious the participant was, and one to tap his/her views regarding the level of support provided to the mentally ill by the community. The original English version of the questionnaire was translated into standard Bengali by a fluent English/Bengali speaker (for use with the older British Bangladeshi participants) and then back-translated (to check for any bias that may have been introduced in the translation).

### Data analysis

The two groups of participants were first compared on demographic variables. In order to reveal the structure of the participants’ attitudes and beliefs about depression, a principle components factor analysis with VARIMAX rotation was conducted across the entire sample for each of the three sections of the questionnaire. To investigate the influence of (i) age and (ii) ethnicity on beliefs about, explanations for, and treatments of depression, multiple regressions were conducted on summated scores for each of the factors identified in the three sections of the questionnaire. Three predictors were used in each regression; age, ethnicity (indicator coded for British Bangladeshi and British White) and an interaction term (age × ethnicity). Prior to each analysis, the age variable was *centred* so that the test between the *intercepts* of the regression lines would provide a test of the difference between the adjusted means for the two ethnic groups – even in the presence of a significant interaction. This method also avoids the loss of statistical power and other problems associated with the dichotomisation of the participants into young and old age groups (MacCallum, Zhang, Preacher, & Rucker, 2002). In cases where the interaction between age and ethnicity was significant, a simple regression was conducted for each ethnic group, with age as the single predictor variable.

## Results

### Demographic information

A summary of the demographic information for the two samples is presented in Table 1.

**Table 1.** Demographic information for the two groups of participants. With the exception of age, the data are in frequencies (%).

Groups	British Bangladeshis (*N* = 190)	British Whites (*N* = 174)
Age in Years: Mean (Range)	28 (17–58)	31 (17–60)
*Gender*		
Male	61 (32)	66 (38)
Female	129 (68)	108 (62)
*Employment*		
Employed^a^	84 (44)	87 (50)
Student	80 (42)	71 (41)
Unemployed	26 (14)	16 (9)
*Marital Status*		
Married	78 (41)	45 (26)
Cohabiting	2 (1)	21 (12)
Divorced/Separated/Widowed	6 (3)	8 (5)
Single	102 (54)	98 (56)
Not Stated	2 (1)	2 (1)
*Religion*		
Christian	0 (0)	120 (69)
Muslim	187 (98)	1 (1)
Other	2 (1)	8 (4)
None/Not Stated	1 (1)	45 (26)
*Piety*		
Very Religious	80 (42)	31 (18)
Moderately Religious	68 (36)	50 (29)
Not Religious	19 (10)	87 (50)
Not Stated	23 (12)	6 (3)
*Educational Qualifications*		
GCSE	30 (16)	19 (11)
A-level	70 (37)	87 (50)
BA/BSc	42 (22)	39 (22)
MA/MSc	14 (7)	7 (4)
PhD	0 (0)	2 (2)
Other	13 (7)	14 (8)
None/Not Stated	21 (11)	6 (3)
Diagnosed with Depression	15 (8)	23 (13)
Knew someone with Depression	100 (53)	121 (70)

Note: ^a^ Includes ‘“housewife”.

There was a small but significant difference between the mean age of the British Bangladeshis and British Whites (*p* < .01). There were no significant differences between the two groups with respect to gender, employment, educational qualifications, and diagnosis of depression. There was a significant difference in marital status (*p* < 0.01) as more British Bangladeshis were married than British Whites, whereas more British Whites were cohabiting than British Bangladeshis. Significant differences in religion and piety existed between the two groups (both *p's* < 0.001). The majority of British Bangladeshi participants were Muslims whereas the majority of British Whites were Christian, and the British Bangladeshis reported being more pious than their British White counterparts. A significant difference was also found with respect to knowing other people who were suffering from depression (*p* < 0.01). A greater proportion of the British White participants than the British Bangladeshi participants reported knowing an individual suffering from depression.

### Factor analyses

The factor analysis conducted on the first section (beliefs about depression) generated a seven-factor solution (eigenvalues > 1.00) and accounted for 56.5% of the total variance. The eigenvalues and variance accounted for by each factor are presented in Table 2.

**Table 2.** The eigenvalues and variance accounted for by each factor in each of the three sections of the questionnaire

	Factors	Eigenvalue	Variance (%)
Beliefs About Depression			56.5
	Myths and Shame	2.59	11.8
	Loss of Dignity	2.15	9.8
	Simplistic Notions	1.80	8.2
	Depression as an Illness	1.67	7.6
	Negative Approach	1.53	6.9
	Helplessness	1.52	6.9
	Sympathy	1.17	5.3
Causal Explanations for Depression			57.2
	Life Events and Illness	4.76	20.7
	Psychological Stress	2.04	8.9
	Biological Explanation	1.91	8.3
	Environment and Aging	1.81	7.9
	Genetics and Substance Abuse	1.37	6.0
	Non-Biological Explanations	1.29	5.6
Treating Depression			57.0
	Religion and Culture	3.81	15.2
	Counselling and Therapy	2.36	11.4
	Medical Treatment	1.68	9.3
	Institutionalisation	1.40	7.2
	Historical Cures	1.12	7.2
	Failure of Psychological Intervention	1.06	6.8

The first factor was labelled *Myths and Shame.* All the items comprising this factor had high mean scores, indicating that participants did not believe that depression is associated with low socioeconomic status, or that the family of a depressed individual should feel shame and hide the depressed individual from the community. Overall, they did not believe that evil spirits cause depression. The second factor was labelled *Loss of Dignity.* Again, the items had relatively high mean scores indicating that the participants did not believe that there is a loss of dignity or respect for the depressive or their family, or that people would not wish to be associated with a depressed individual. The third factor was labelled *Simplistic Notions.* Participants did not think that depression was the same thing as being unhappy, or that simply suggesting that the depressed individual should go out to buy new clothes would be useful. They were more equivocal regarding the efficacy of talking to a family member rather than a friend. The fourth factor we labelled *Depression as an Illness.* Although participants believed that depression was a mental illness, they were more equivocal about the notion that depression runs in families. Perhaps surprisingly, that were also equivocal about the notion that seeking help from a psychiatrist was better than trying to deal with the problem oneself. The next factor contained just two items, and was labelled *Negative Approach.* The mean ratings indicate that in general, the participants felt that talking to a mental health professional would be useful, and that something could be done to help the depressed individual. The sixth factor was labelled *Helplessness*. Participants believed that depressed people lose control over important things in their lives, but also thought that it would be helpful for the depressed person to engage in an activity that he/she enjoyed. The final factor was *Sympathy*. An inspection of the mean scores indicates that participants thought that one could sympathise with a depressed person, and they believed that the risk of depression was reduced if an individual discussed their problems with someone. However, they were more equivocal with regard to the sympathy shown towards depressed individuals by the community.

The factor analysis conducted on the second section of the questionnaire (causal explanations for depression) produced a six-factor solution (eigenvalues > 1.00), and accounted for 57.2% of the total variance. The eigenvalues and variance accounted for by each factor are presented in Table 2. The first factor was labelled *Life-events and Illness.* This factor comprised a wide range of items all of which had low mean scores, indicating that participants believed that social factors, financial stresses, traumatic experiences and physical illness could all lead to the onset of depression. Scores for the items making up the second factor, labelled *Psychological Stress*, suggest that the participants believed psychological stress as a result of social isolation, loss of occupation or general social pressure could lead to depression. The third factor, *Biological Explanation*, had mean scores relatively close to the centre of the 6-point scale. Thus participants seemed equivocal with respect to the notions that depression is caused either by an imbalance of chemicals or hormones in the body, or that people become depressed for no apparent reason. The fourth factor, *Environment and Aging*, also had mean scores close to the middle of the scale. Participants adopted a neutral position with respect to the role of society, the weather, and aging as causes of depression. The fifth factor, *Genetics and Substance Abuse*, indicates that participants believed that substance abuse could be a source of depression, but rejected the association of blood-ties with depression. The final factor, *Non-Biological Explanations*, indicated that participants did not believe that depression had a supernatural origin, but were equivocal with respect to depression resulting from a boring, mundane lifestyle.

The factor analysis on the third section of the questionnaire (treating depression) again generated a six-factor solution (eigenvalues > 1.00) and accounted for 57.0% of the total variance. The eigenvalues and variance accounted for by each factor are presented in Table 2. The first factor was labelled *Religion and Culture.* Participants generally believed the family, friends, and religion were important factors in the treatment of depression. Moreover, the participants believed the society and self-help could also play a role in relieving depression. They were far less sure that trying to think positively or focusing attention elsewhere would cure depression. The second factor was labelled *Counselling and Therapy.* The means scores on the questions making up this factor indicate that the participants generally believed that psychological intervention (therapist, counsellor or self-help group) was useful in the treatment of depression. The third factor was *Medical Treatment.* The mean scores suggested that participants were rather equivocal with respect to the role of physicians and antidepressants in the treatment of depression. The means scores for the fourth factor, *Institutionalisation*, indicated that participants were again equivocal with respect to the stigmatising and negative effects of mental institutes on patients. The fifth factor, *Historical Cures* contained just two items. Participants did not believe that faith healers could cure depression, nor did they think that the mentally ill should be kept in hospital until they completely recover. The last factor, *Failure of Psychological Intervention*, contained a single item. Participants indicated that they were unsure that a psychological intervention could cure depression.

### Cross-cultural comparisons

The regression results for the first section of the questionnaire, beliefs about depression, are presented in Table 3 – together with the adjusted means for the two ethnic groups.

**Table 3.** The regression results for the seven factors identified in the first section of the questionnaire (Beliefs About Depression)

				β			Adjusted Means^a^	
Factor	*F*-ratio	*dfs*	*R*^2^ (%)	Age	Ethnicity	Age × Ethnicity	British Bangladeshis	British Whites
Myths and Shame	43.99***	3, 356	27.0	−.45***	.47***	.37***	4.61	5.51
Loss of Dignity	28.11***	3, 358	19.1	−.56***	.31***	.31***	3.94	4.70
Simplistic Notions	28.49***	3, 352	19.5	−.35***	.41***	.21**	3.85	4.68
Depression as an Illness	9.60***	3, 355	7.5	−.29***	−.17**	.34***	3.31	2.96
Negative Approach	7.22***	3, 354	5.8	−.22**	.21***	.15	4.41	4.92
Helplessness	10.21***	3, 351	8.0	−.14	.28***	.13	2.87	3.41
Sympathy	0.06	3, 352	0.0	−.01	.02	.01	3.00	3.04
Notes: *p < .05.								
**p < .01.								
***p < .001.								
^a^ Adjusted for age and age *×* ethnicity.						

Notes: *p < .05.

** *p* < .01.

*** *p* < .001.

a Adjusted for age and age × ethnicity.

For the first factor, *Myths and Shame*, there was a significant effect of age, ethnicity, and the interaction between these two variables. The British Bangladeshi group had a lower adjusted mean score on this factor than the British Whites, indicating that the British Bangladeshi participants were more likely to believe that there is a sense of shame associated with depression, that depression is more common in the lower socioeconomic classes, and that a depressed individual may be possessed by evil. However, it should be noted that both adjusted means are well above the mid-point of the 6-point Likert-like scale. The simple regressions revealed a significant negative relationship between age and summated score for the British Bangladeshi participants, *β* = −.34, *p* < .001, indicating that beliefs concerning shame, socioeconomic status, and evil spirits were more prevalent with increasing age within this group. There was no age effect for the British White participants, *β* = .04, *p* = .614. For the second factor, *Loss of Dignity*, there was again an effect of age and ethnicity, and an age × ethnicity interaction. The British Bangladeshi participants had a significantly lower adjusted mean score than the British White participants on this factor, indicating that they were more likely than the British Whites to believe that a depressed individual and his or her family lose respect and dignity. However, the mean scores for both groups were above the mid-point of the scale. The simple regressions revealed that within the British Bangladeshis, a belief in loss of dignity was stronger with increasing age, *β* = −.44, *p* < .001, but no such trend was found within the British White group, *β* = −.09, *p* = .245. For the third factor, *Simplistic Notions*, there was a significant effect of both age and ethnicity – and a significant age × ethnicity interaction. The adjusted mean score for the British Bangladeshi group was significantly lower than for the British White group. Thus the British Bangladeshi participants were more likely than the British Whites to believe that being depressed is the same as being unhappy, that talking to family members can help, and that the depressed individual can be helped by simply going out to buy new clothes. A simple regression showed that within the British Bangladeshi group, increasing age was associated with stronger beliefs in the simplistic notions making up this factor, *β* = −.28, p < .001. This trend was not evident in the British White group, *β* = −.12, *p* = .106. For the fourth factor, *Depression as an Illness*, there was a significant effect of age, ethnicity and a significant interaction. The adjusted mean score on this factor for the British Whites was significantly lower than for the British Bangladeshis. Thus, overall, the British Whites were more likely to view depression as being an illness than were the British Bangladeshi participants. However, the simple regressions reveal that within the British Bangladeshi group, older participants were *more* likely to view depression as an illness, *β* = −.23, *p* = .002, whereas within the British White group, older participants were *less* likely to view depression as an illness, *β* = .18, *p* = .018. For the fifth factor, *Negative Approach*, there were significant effects of both age and ethnicity, but no interaction. Older participants were more likely to believe that depression is *not* helped by talking to a psychologist or psychotherapist, and that there is probably little that depressed individuals can do about their condition. The ethnicity main effect indicates that these views were stronger in the British Bangladeshi group than in the British White group. For the sixth factor, *Helplessness*, the overall regression was significant, but of the individual predictors, only ethnicity reached significance. The British Bangladeshi participants were more likely to believe that depression is associated with helplessness and loss of control than the British White participants. For the seventh factor, *Sympathy*, neither the overall regression nor any of the individual predictors were significant.

The regression results for the second section of the questionnaire, causal explanations for depression, are presented in Table 4.

**Table 4.** The regression results for the six factors identified in the second section of the questionnaire (Causes Explanations for Depression)

					β			Adjusted Means^a^	
Factor	*F*-ratio	*dfs*	*R*^2^ (%)	Age	Ethnicity	Age × Ethnicity	British Bangladeshis	British White
Life Events and Illness	3.33*	3, 348	2.8	.25**	−.04	−.11	2.36	2.30
Psychological Stress	9.04***	3, 352	7.2	.07	.11*	.17	2.42	2.63
Biological Explanations	17.63***	3, 358	12.9	−.03	−.35***	.12	3.62	2.85
Environment and Aging	7.27***	3, 355	5.8	.19*	−.17**	.00	3.59	3.26
Genetics and Substance Abuse	9.90***	3, 356	7.7	.39***	−.07	−.45***	2.66	2.51
Non-Biological Explanations	32.31***	3, 357	21.4	.01	.46***	.01	3.33	4.40

Notes: *p < .05.

** *p* < .01.

*** *p* < .001.

a Adjusted for age and age × ethnicity.

The first factor in this section was *Life Events and Illness.* There was a significant age effect for this factor, but no significant effect of ethnicity and no interaction. The results indicate that older participants (in both groups) were less likely to believe that social factors, financial stresses, traumatic experiences and physical illness could lead to the onset of depression. However this effect was weak – accounting for only 2.8% of the total variance. For the second factor, *Psychological stress*, the overall regression was significant, but of the individual predictors, only ethnicity reached significance. The British Bangladeshis were more likely to believe that psychological stress could cause depression than were the British White participants. For the third factor, *Biological Explanations*, there was no effect of age, a highly significant effect of ethnicity, and no interaction. The adjusted mean score for the British White participants was significantly lower than for the British Bangladeshis. Thus the British Whites were more likely to believe in a biological explanation for depression than were the British Bangladeshi participants. For the fourth factor, *Environment and Aging*, there was a significant effect of age and ethnicity – but no interaction between these variables. The adjusted mean score for the British White participants was significantly lower than for the British Bangladeshi participants. These results suggest that British Bangladeshis were less likely than the British Whites to believe in an environmental cause for depression, such as the weather or society, or that aging per se can cause depression; and older participants were less likely to believe in these causes than younger participants. However, both effects were weak; the age effect accounted for only 1.4%, and the ethnicity effect 2.8%, of the total variance. For the fifth factor, *Genetics and Substance Abuse*, there was a significant effect of age, no effect of ethnicity, but a highly significant cross-over interaction between age and ethnicity. The simple regression for the British Bangladeshi group revealed that the older participants were *more* likely to believe that depression is caused by having blood relatives who are depressed and *less* likely to believe that depression is caused by drug abuse, *β* = .30, *p* < .001. The opposite pattern was found for the British White group; older participants were less likely to believe that a blood relationship was of importance, and were *more* likely to believe that depression could result from drug abuse, *β* = −.22, *p* = .004. The final factor in this section was *Non-Biological Explanations.* For this factor, there was a significant effect of ethnicity, but no effect of age and no interaction between age and ethnicity. The mean score on this factor for the British White participants was significantly lower than for the British Bangladeshi participants. Thus, the British Whites were less likely to believe that depression could result from black magic, or a mundane life, than were the British Bangladeshi participants.

The regression results for the third section of the questionnaire, treating depression, are presented in Table 5.

**Table 5.** The regression results for the six factors identified in the third section of the questionnaire (Treating Depression)

					β			Adjusted Means^a^	
Factor	*F*-ratio	*dfs*	*R*^2^ (%)	Age	Ethnicity	Age × Ethnicity	British Bangladeshis	British Whites
Religion and Culture	25.98***	3, 350	18.2	.09	.42***	−.11	2.53	3.21
Counselling and Therapy	5.38**	3, 355	4.4	−.09	−.18***	.00	2.91	2.60
Medical Treatment	7.45***	3, 352	6.0	−.39***	.03	.24**	3.47	3.52
Institutionalisation	3.71*	3, 357	3.0	.20*	−.12*	−.08	3.54	3.24
Historical Cures	39.93***	3, 359	25.0	−.46***	.45***	.37***	3.97	5.01
Failure of Psychological Intervention	0.96	3, 359	0.8	.03	.06	−.09	3.29	3.44

Notes: *p < .05.

** *p* < .01.

*** *p* < .001.

a Adjusted for age and age × ethnicity.

For the first factor in this section, *Religion and Culture*, there was a significant ethnicity effect but there was no effect of age and no interaction. The result indicates that British Bangladeshi participants placed greater importance on religion, family, friends, self-help and society in the treatment for depression than did the British White participants. The overall regression was significant for the second factor, *Counselling and Therapy*, but of the individual predictors, only ethnicity reached significance. This indicates that the British Bangladeshi group had less faith in the effectiveness of counselling and therapy in the treatment of depression than the British White group. For the third factor, *Medical Treatment*, there was a significant effect of age – no effect of ethnicity – but a significant age × ethnicity interaction. As with the *Genetics and Substance Abuse* factor in the second section of the questionnaire, the interaction was cross-over in nature. The simple regression for the British Bangladeshi group revealed that a belief in medical (as distinct from psychological) treatment for depression *increased* with increasing age, *β* = −.32, p < .001, but there was no such trend in the British White group, *β* = −.10, *p* = .201.

For the fourth factor, *Institutionalisation*, there were weak but significant effects of age and ethnicity, but no interaction. The results indicate that older participants in both groups were less likely to think that being in a mental hospital is stigmatising and has as a negative effect on the patient than the younger participants, but overall these beliefs were stronger amongst British Whites than British Bangladeshis. However, age accounted for only 1.5%, and ethnic group for only 1.4%, of the total variance. For the fifth factor, *Historical Cures*, there was a significant effect of age, ethnicity, and an age × ethnicity interaction. The adjusted mean score for the British Bangladeshi group was significantly lower than for the British White group. A simple regression revealed a significant age effect for the British Bangladeshi participants, *β* = −.32, *p* < .001, indicating that older British Bangladeshis were more likely than younger British Bangladeshis to believe that patients should be kept in mental hospitals until they are cured, and that a faith healer can cure depression. There was no effect of age in the British White group, *β* = .01, *p* = .934. For the sixth and final factor in this section, *Failure of Psychological Intervention*, there were no effects of age or ethnicity, and no interaction.

## Discussion

Overall, the findings suggest that there are differences in attitudes and beliefs about depression between British Bangladeshis and British Whites, and that these differences are often moderated by age – particularly within the Bangladeshi population. We note that there appears to be very little difference between the two cultures in the young age group with respect to knowledge of depression.

The older British Bangladeshis generally tended to show more negative attitudes towards depression than younger British Bangladeshis and British Whites. Thus attitudes towards depression were moderated by age within the Bangladeshis but not within the British Whites, where both young and old generally demonstrated similar attitudes towards depression. The results are generally consistent with other studies in the area (e.g., Gonzalez et al., 2011; Peluso & Blay, 2009).

With respect to *general beliefs* about depression, and unlike younger British Bangladeshis and British Whites, older British Bangladeshis associate depression with personal shame, and loss of respect and dignity for the family. This finding is consistent with the findings from the study by Furnham and Malik (1994) and supports the general findings that Asians having more stigmatising attitudes towards mental illness than Whites. The older British Bangladeshis also found it hard to sympathise with a depressive and also believed the community to be less sympathetic and understanding, implying that the depressed person is not valued by society. British Bangladeshis are more likely than British Whites to believe that being depressed is the same as being unhappy and this view also increases with age. They are also more likely to view depression as an illness with increasing age whereas the reverse is true for British Whites. British Bangladeshis are more likely to associate depression with helplessness and loss of control than are British Whites and this view is not moderated by age. Finally, older individuals in both ethnic groups are less likely than younger individuals to believe that psychotherapy is useful in the treatment of depression, and more likely to think that there is little a depressed individual can do about his or her condition.

With respect to *causal explanations* for depression, older individuals in both groups are less likely to believe that depression can be caused by exogenous factors (e.g., environmental or social factors, financial stresses, traumatic experiences or physical illness). However, British Bangladeshis are more likely to believe that depression can be caused by psychological stress and less likely to believe it can be caused by biological factors than British Whites. They are less likely to think that depression is caused by environmental factors or ageing *per se*, and more likely to believe it has a genetic basis than British Whites – although they are also more likely to believe that depression can have a supernatural aetiology – and that faith healing can cure depression. An interaction between cultural and age was found; within British Bangladeshis older participants are more likely to believe that depression has a genetic component and less likely to believe that depression is caused by drug abuse than younger individuals, whereas the *opposite* pattern is evident amongst the White British.

With respect to *treatment* of depression, British Bangladeshis place greater importance on the role of religion, family and friends, and have less faith in the effectiveness of counselling and therapy than British Whites. This was expected, and highlights the importance of the family in the British Bangladeshi culture and supports the notion that Asians are more likely to use a lay referral system rather than seeking professional help. This may imply that the British Bangladeshis do not view depression to be such a serious illness, and the depressed individual can get help from the family rather than a health professional, but alternatively, it may indicate a fear of being stigmatised. Older British Bangladeshis are more likely to believe in the efficacy of medical as opposed to psychological intervention than younger British Bangladeshis or British Whites, but they are also willing to seek help from other sources such as faith healers. Finally, older individuals in both groups are less likely than younger individuals to think that being in a mental hospital is stigmatising, and older British Bangladeshis are more likely to believe that a stay in a mental hospital can cure depression.

This study has implications for the provision of appropriate psychiatric facilities for migrant communities, whose values are influenced by traditional cultural norms. It is important not to over-generalise cultural stereotypes since there is distinct diversity in values and attitudes within these cultures. Age was found to have significant impacts on the general beliefs and attitudes towards depression. Presumably therefore an older Bangladeshi's pathway to treatment or advice/help that they may give others will be different from younger Bangladeshi's. This pattern may be equally true of other immigrant groups where older migrants are more representative of the culture from which they come than the younger generation who take on the predominant beliefs found in the majority host culture.

The clinical implications are primarily concerned with the mental health literacy of older Bangladeshi's who may not recognise the signs and symptoms of various types of depression in themselves, family and acquaintances. It may be therefore, particularly important to target this group with information and assistance. Indeed it seems that Islamic religious leaders may play an important part in this, particularly when they are seen to support and agree with health care professionals. Whilst there seems to be tension between Islam and certain types of faith healers, the role of Islam in the early development of medicine could be emphasised so help older migrants accept the advice of health care professionals with respect to their and others symptoms of depression and they way to get effective help.
